# Transport of organic anions and cations in murine embryonic kidney development and in serially-reaggregated engineered kidneys

**DOI:** 10.1038/srep09092

**Published:** 2015-03-13

**Authors:** Melanie L. Lawrence, C-Hong Chang, Jamie A. Davies

**Affiliations:** 1 Centre for Integrative Physiology, The University of Edinburgh

## Abstract

Recent advances in renal tissue engineering have shown that dissociated, early renogenic tissue from the developing embryo can self-assemble into morphologically accurate kidney-like organs arranged around a central collecting duct tree. In order for such self-assembled kidneys to be useful therapeutically or as models for drug screening, it is necessary to demonstrate that they are functional. One of the main functional characteristics of mature kidneys is transport of organic anions and cations into and out of the proximal tubule. Here, we show that the transport function of embryonic kidneys allowed to develop in culture follows a developmental time-course that is comparable to embryonic kidney development *in vivo*. We also demonstrate that serially-reaggregated engineered kidneys can transport organic anions and cations through specific uptake and efflux channels. These results support the physiological relevance of kidneys grown in culture, a commonly used model for kidney development and research, and suggest that serially-reaggregated kidneys self-assembled from separated cells have some functional characteristics of intact kidneys.

The mature vertebrate kidney is a highly specialised and complex excretory organ, consisting of many different cell types, with numerous physiological functions. Murine kidney development begins with formation of a ureteric bud as a branch from the nephric duct at the caudal end of the embryo. At embryonic day 11.5 (E11.5), the rudimentary kidney is composed of two distinct tissue types, the epithelial ureteric bud surrounded by condensed mesenchyme. Previous work in this laboratory has shown that kidney rudiments at this stage can be dissociated into a single cell suspension, and these cells can self-assemble into organotypic renal structures^1^. Refinement of this technique by serial reaggregation produces “engineered” kidneys that are very similar, morphologically, to intact kidneys grown in culture^2^, and further refinement using the Sebinger culture method^3^ allows development of physiologically relevant structures such as loops of Henle^4^. Although serially-reaggregated embryonic kidneys are morphologically similar to kidneys developing intact in culture, it is not known if these kidneys are functional in the same way as intact cultured embryonic kidneys, or as embryonic kidneys *in vivo*. The ability of separated kidney progenitor cells to self-assemble into functional kidney-like rudiments centred around a single collecting duct would be of great importance both to regenerative medicine and for use of cultured or engineered kidneys as a drug screening platform.

The nephrons of mature kidneys in vertebrates represent one of the major excretory routes in the body. Specialised transporters and efflux pumps at the basolateral and apical sides of the proximal tubules mediate the transport of organic anions and cations, providing a mechanism for excretion of xenobiotics and endogenous compounds (reviewed in ref. 5). The functional properties of proximal tubules in adult kidneys have been studied in many species including human, mouse and rat, with varying degrees of species differences reported. For example, in humans the primary tubular cation transporter in the kidney is OCT2, located in the proximal tubules, with OCT1 expression mainly localised to hepatocytes and brain^6^ whereas, in rodents, both Oct1 and Oct2 are expressed in the proximal tubules of the kidney and both mediate uptake of organic cations^7^,^8^.

Although much is understood about tubular excretory processes in mature animals, less is known about the development of this transport in the embryo. In rats, transcripts for organic anions transporters (Oats) and organic cation transporters (Octs) have been shown to be detectable in embryonic stages both *in vivo* and in culture^9^,^10^, with the transcripts for anion transporters appearing earlier than cation transporters^10^. Uptake of fluorescent anion has also been shown in cultured rodent kidneys at later stages of development and in cultured recombined rodent kidney tissue^9^,^10^,^11^,^12^. It has also been shown that transcript levels for ion transporters *in vivo* increase gradually during development to birth, and continue to increase postnatally until adulthood^13^,^14^,^15^. However, the functional development of transport (both uptake and efflux) from a temporal aspect has not been previously reported *in vivo*, either in cultured rodent embryonic kidneys, or in serially-reaggregated engineered kidneys. Understanding the temporal and spatial development of tubular function is important for our understanding of nephron development in embryogenesis, and for understanding the extent to which serially-reaggregated engineered kidneys, and murine embryonic kidneys in culture, may be physiologically useful and relevant as a model system.

Using the Sebinger culture system, which encourages cultures to grow flat whilst preserving development of renal structures, we have optimised and developed a panel of live fluorescence-based assays to test tubular function in cultured embryonic kidneys and in serially-reaggregated, engineered kidneys. We find that, in agreement with transcript data from rat embryonic kidneys *in vivo*^10^, development of anion uptake function in mice precedes cation uptake both in cultured kidneys and *in vivo* and that efflux of anions through the Mrp family of efflux pumps occurs at a later stage than anion uptake. We also find that cultured kidneys dissected at E11.5 and placed in culture continue to develop functional characteristics at roughly the same rate as kidneys allowed to develop *in vivo*, supporting their validity as a relevant model for kidney research. Furthermore, we find that the tubules of serially-reaggregated engineered kidneys are able to transport anions and cations in a manner similar to the mature kidney, supporting their possible use therapeutically or for pharmacological testing.

## Results

### Development of assays to test tubular uptake and efflux of organic ions in cultured embryonic kidneys

**Figure 1:**
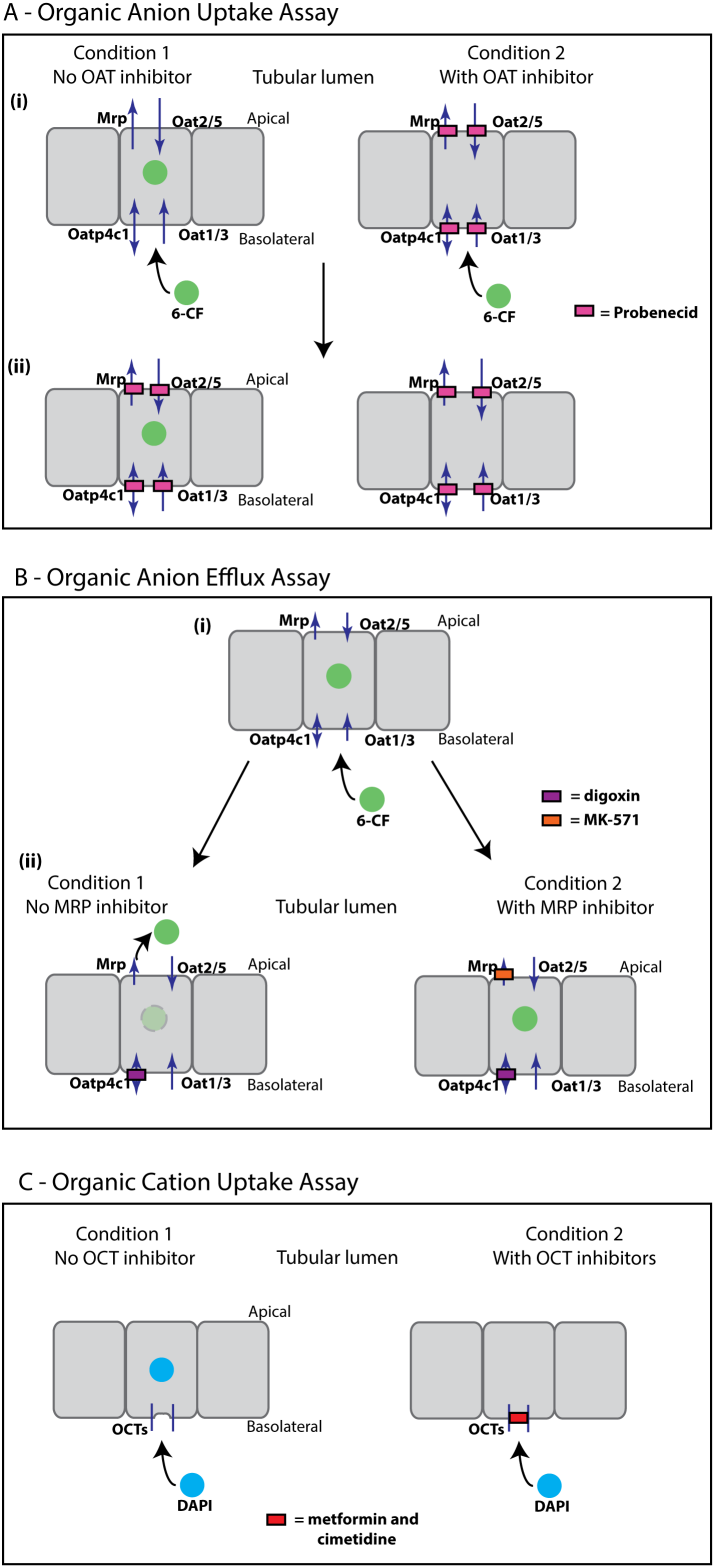
Transport assays for live whole embryonic kidney culture. Arrows indicate direction of transport, filled boxes indicate inhibition of transport. (A) Organic Anion Uptake Assay – (i) The fluorescent anion 6-carboxyfluorecein (6-CF) is taken up in the tubules of cultured kidneys by organic anion transporters. Cultured kidneys are either treated with 6-CF only, or 6-CF plus Oat inhibitor (probenecid, 2.5 mM), and uptake is allowed to progress for one hour. (ii) Live cultured kidneys are imaged immediately following treatment of both conditions with probenecid (10 mM) to inhibit efflux of 6-CF and retain fluorophore intracellularly. (B) Organic Anion Efflux Assay – The fluorescent anion 6-CF is taken up by the tubules of cultured kidneys and then exported into the lumen of the tubules through the Mrp family of efflux pumps and possibly by other routes. (i) Cultured kidneys are treated with 6-CF for one hour to allow uptake and then (ii) treated with either digoxin alone (to block Pgp, BCRP and Oatp4c1), or digoxin plus MK-571, a specific Mrp-family inhibitor. For clarity only the Mrp family is shown. (C) Organic Cation Uptake Assay – The fluorescent cation DAPI is taken up in the tubule cells of cultured kidneys by organic cation transporters. Cultured kidneys are either treated with DAPI, or with DAPI plus Oct inhibitors (metformin and cimetidine). All assays are with live cultured embryonic kidneys and imaging is carried out on live kidneys or serial reaggregates.

In order to investigate the emergence of tubular function in renal development, we developed and optimised a series of assays to test tubular transport of organic anions and cations in live, cultured, developing murine kidneys. These assays could then be used to test transport function in the serially-reaggregated, engineered kidneys. Specific inhibitors of the different transport channels were employed to demonstrate specificity of fluorescent cation uptake and fluorescent anion uptake or efflux. The assays (anion uptake, anion efflux and cation uptake) are summarised in Figure 1.

#### Assay for Organic Anion Uptake

The fluorescent anion 6-carboxyfluorescein (6-CF) was chosen as a tracer dye to investigate organic anion transport in cultured embryonic kidneys and in serially-reaggregated engineered kidneys. The 376 Dalton fluorescein derivative 6-CF has been shown to be transported by organic anion transporters (Oats)^9^,^10^,^11^,^12^. Uptake of organic anions in mice is mediated by organic anion transporters localized to both the basolateral (Oat1/3 and Oatp4c1) and apical surfaces (Oat2/5)^5^,^16^,^17^. In addition, Urat1 is located at the apical surface of proximal tubules: Urat1 is, however, a urate-anion exchanger and is therefore not likely to be relevant to cultured kidneys because cultured kidneys are not filtering blood or regulating serum urate levels. 6-CF is also likely to be transported by the basolaterally-located Oatp4c1^18^, a member of the organic anion transport peptide (Oatp) family of anion transporters, which preferentially transport anions over 350 Daltons^16^.

Probenecid is a potent and broad inhibitor of anion transporters, including the murine basolateral renal tubular Oat family of anion transporters^19^, and the apical Oat2 and Oat 5^20^,^21^. Since probenecid inhibits other members of the Oatp family^22^ it may also be an inhibitor of Oatp4c1, although this has not been specifically reported to our knowledge. In addition, probenecid has been found to inhibit the apically-located Mrp-family of efflux pumps^23^,^24^.

Anion uptake was assessed by incubating live, cultured kidneys growing in the Sebinger culture system with the fluorescent anion 6-CF, either with or without probenecid (2.5 mM)^19^ (Figure 1A). After allowing uptake to proceed for one hour, both conditions were treated with a higher concentration of probenecid (10 mM) to block efflux of 6-CF by the Mrp-family of efflux pumps, and thus to trap sufficient fluorophore intracellularly for imaging purposes (adapted from methods by Rosines et al.^12^, and Rak-Raszewska et al.^11^).

#### Assay for Organic Anion Efflux

Efflux of anions into the lumen of the proximal tubules is mediated by the Mrp family and Breast Cancer Resistance Protein (Bcrp), and to some extent by P-glycoprotein (Pgp, Mdr1). Although the basolateral Oatp4c1 is thought to mediate uptake of anions into the cell^18^, it is also possible that it may mediate basolateral efflux as many of the Oatp family are capable of bidirectional transport^16^.

Fluorescein efflux has been shown to be mediated by the Mrp family of luminal efflux pumps^25^,^26^, which are inhibited by the Mrp family-specific inhibitor MK-571^27^. Although efflux of fluorescein or its derivative 6-CF has not specifically been shown to be mediated by Pgp, Bcrp or Oatp4c1, we decided to inhibit these routes of efflux to isolate transport by the Mrp family of efflux pumps.

Digoxin is known to be transported by Oatp4c1^18^, and effluxed apically into the lumen of the renal tubules by P-glycoprotein (Mdr1)^28^. Its use as a competitive inhibitor of Oatp4c1 and Bcrp transport channels has also been reported^18^,^29^. On the other hand, transport of digoxin by Mrp is minimal in comparison^30^.

Anion efflux at the apical side was again assessed using the fluorescent anion 6-CF. For the efflux assay, both conditions were allowed to take up 6-CF through the organic anion transporters (Figure 1B (i)). After allowing uptake of the fluorescent anion 6-CF, live kidneys in Sebinger culture were washed and treated with digoxin to inhibit efflux through channels other than Mrps. Treatment with digoxin was either with or without the Mrp-family-specific inhibitor MK-571^27^ in order to test the specificity of anion efflux by this efflux pump in cultured and engineered kidneys (Figure 1B (ii)).

#### Assays for Organic Cation Uptake

In addition to anion uptake and efflux, we investigated the ability of the proximal tubules of cultured embryonic kidneys to take up organic cations using the fluorescent cationic molecule 4',6-diamidino-2-phenylindole (DAPI) (Figure 1C). DAPI is not normally permeable to live cells with intact cell membranes, however, the dye can be transported into epithelial cells via Oct1, which is expressed in the kidney in mice^31^. Uptake of DAPI by Octs was assessed either with or without the organic cation transporter family inhibitors metformin and cimetidine^32^,^33^,^34^.

These assays were used as the basis for analysing tubular function in cultured kidneys and serially-reaggregated engineered kidneys.

### Onset of tubular uptake and efflux of anions by organic anion channels in cultured embryonic kidneys occurs in a temporally and physiologically relevant manner

**Figure 2:**
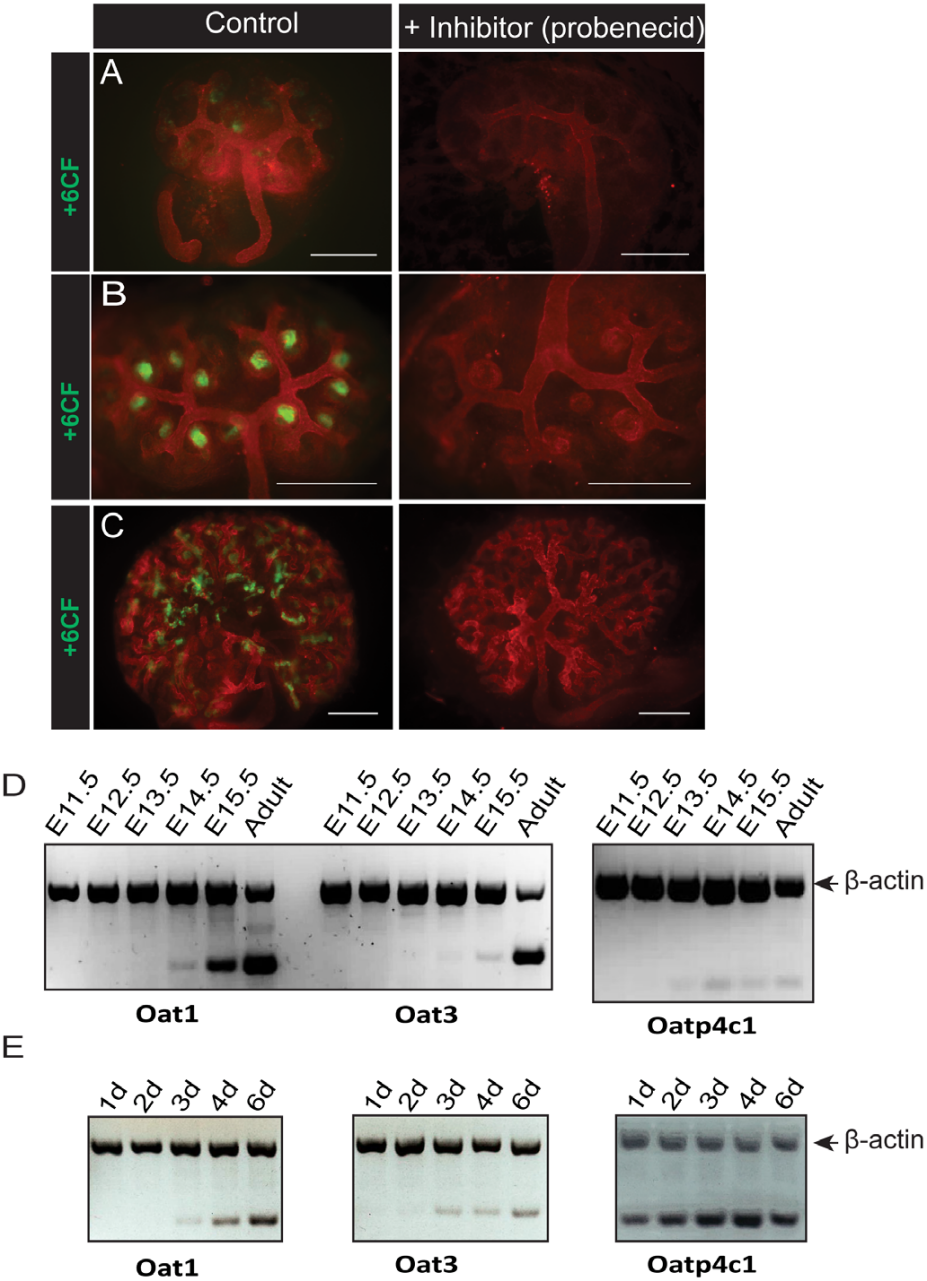
Onset of tubular organic anion uptake in developing kidneys in culture and in vivo. Specific uptake of the fluorescent anion 6CF with or without the Oat inhibitor probenecid was investigated in E11.5 mouse kidneys grown in Sebinger culture after 1 day (A), 2 days (B) or 6 days (C) in culture. Scale bars represent 300 μm. (D) Total mRNA was extracted from kidneys at various embryonic stages and from adult murine kidney. Detection of mRNA transcripts from three renal organic anion transporters was evaluated by multiplex RT-PCR (gene of interest and β-actin). (E) Total mRNA was extracted from E11.5 embryonic kidneys cultured for 1, 2, 3, 4, or 6 days. Detection of mRNA transcripts from three renal organic anion transporters was evaluated by multiplex RT-PCR (gene of interest and β-actin).

We first assessed qualitatively the ability of embryonic kidneys, cultured from E11.5, to take up the fluorescent anion 6-CF specifically in the presence or absence of probenecid, a well-characterised Oat inhibitor. We found that cultured embryonic kidneys are able to transport organic anions early in development (Figure 2). The specific function of anion transporters appeared as early as 2 days in culture (Figure 2B), at the earliest stage of nephron formation. This functional aspect of renal tubules was maintained throughout the culture of the embryonic kidneys, with increasing numbers of developing nephrons exhibiting specific tubular uptake (Figure 2C). As expected, there was no evident uptake of the fluorescent anion 6-CF after 1 day in culture, when nephrons are only beginning to be induced (Figure 2A).

In order to compare the functional onset of anion transport in kidneys in Sebinger culture to kidneys allowed to develop *in vivo*, we analysed transcripts for the main renal anion transporters at different embryonic time-points, using semi-quantitative multiplex RT-PCR (Figure 2D). In addition, we also analysed transcripts from cultured, E11.5 embryonic kidneys that were grown in culture for varying lengths of time. Transcripts for two of the renal anion transporters, Oat1 and Oat3^35^,^36^, were detected at embryonic day 14.5 (E14.5), while transcript for the basolateral anion transporter Oatp4c1 was detected from E13.5. In cultured kidneys, transcripts for Oat1 and Oat3 were detected after 3 days in culture, whilst transcript analysis for Oatp4c1 showed detectable transcript from the first day in culture.

We next assessed the ability of cultured embryonic kidney tubules to export organic anions apically into the tubular lumen. After allowing the fluorescent anion 6-CF to be taken up by the tubules in cultured embryonic kidneys, efflux by the Mrp family was assayed by treating the live kidneys with digoxin (to eliminate other possible routes of anion efflux), either with or without the Mrp inhibitor MK-571^27^.

**Figure 3:**
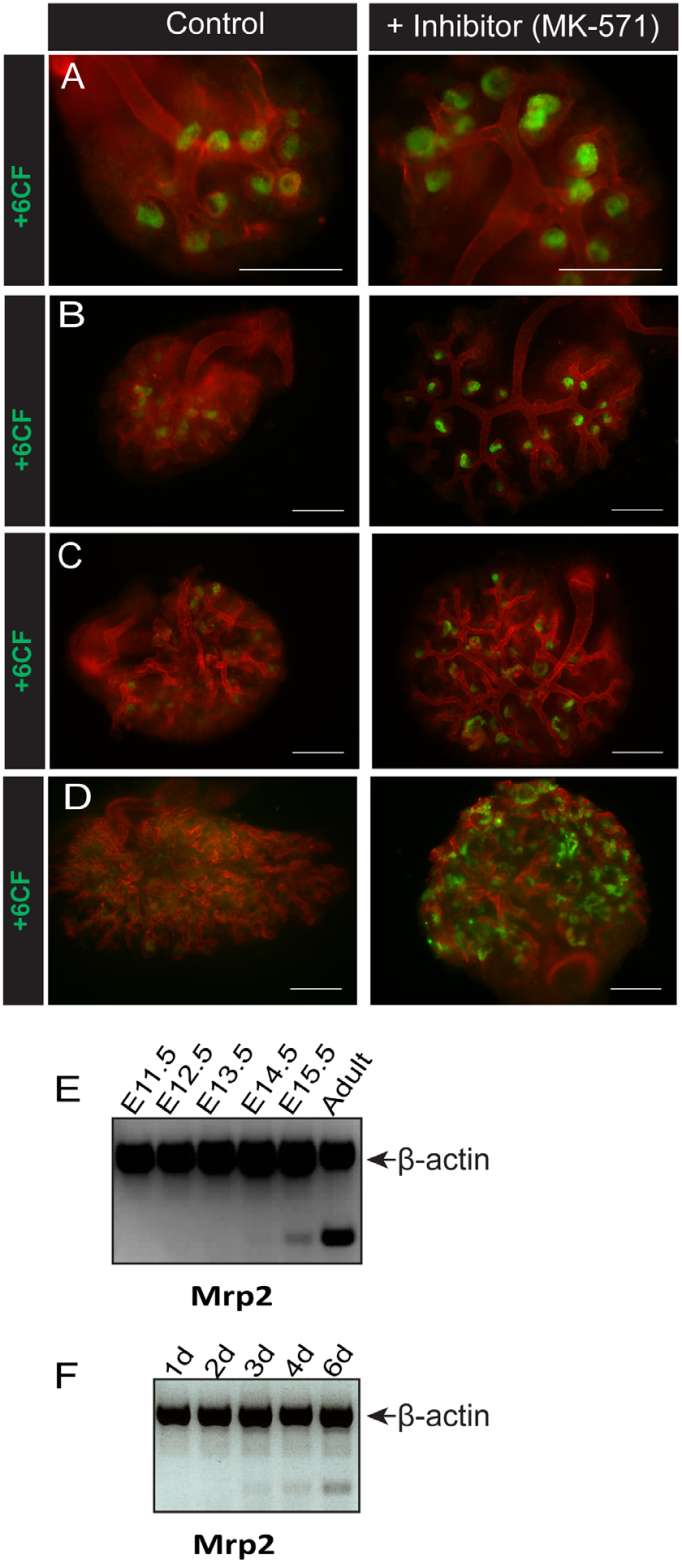
Onset of tubular organic anion efflux in developing kidneys in culture and in vivo. Specific efflux of the fluorescent anion 6CF with or without the Mrp family-specific inhibitor MK-571 was investigated in E11.5 mouse kidneys grown in Sebinger culture after 2 days (A), 3 days (B), 4 days (C) or 6 days (D) in culture. Scale bars represent 300 μm. (E) Total mRNA was extracted from kidneys at various embryonic stages and from adult murine kidney. Detection of mRNA transcripts from one of the main renal Mrp efflux pumps (Mrp2) was evaluated by multiplex RT-PCR (gene of interest and β-actin). (F) Total mRNA was extracted from E11.5 embryonic kidneys cultured for 1, 2, 3, 4, or 6 days. Detection of mRNA transcripts from Mrp2 was evaluated by multiplex RT-PCR (gene of interest and β-actin).

No efflux of anions by cultured embryonic kidneys was observed (Figure 3A) before 3 days in culture, from when it increased (Figure 3B). After 4 days in culture, efflux was clearly apparent except in kidneys treated with MK-571 (Figure 3C), and efflux mediated specifically by Mrp transport was maintained after 6 days in culture and beyond (Figure 3D).

An important Mrp-family efflux pump expressed in the murine kidney is Mrp2. Transcript analysis of Mrp2 by semi-quantitative multiplex RT-PCR in embryonic kidneys allowed to develop *in vivo* and dissected at different embryonic time-points indicated that the transcript for Mrp2 can be clearly detected at E15.5 (Figure 3E). In cultured kidneys, the Mrp2 transcript is apparent from about 3–4 days in culture (Figure 3F).

### Onset of tubular uptake of cations by organic cation channels in cultured embryonic kidneys also occurs in a temporally and physiologically relevant manner

Proximal tubule epithelial cells in the kidney in mice also take up organic cations. The onset of tubular function with respect to uptake of cations in the developing murine kidney and the extent to which this function is developed in culture has not been reported.

**Figure 4:**
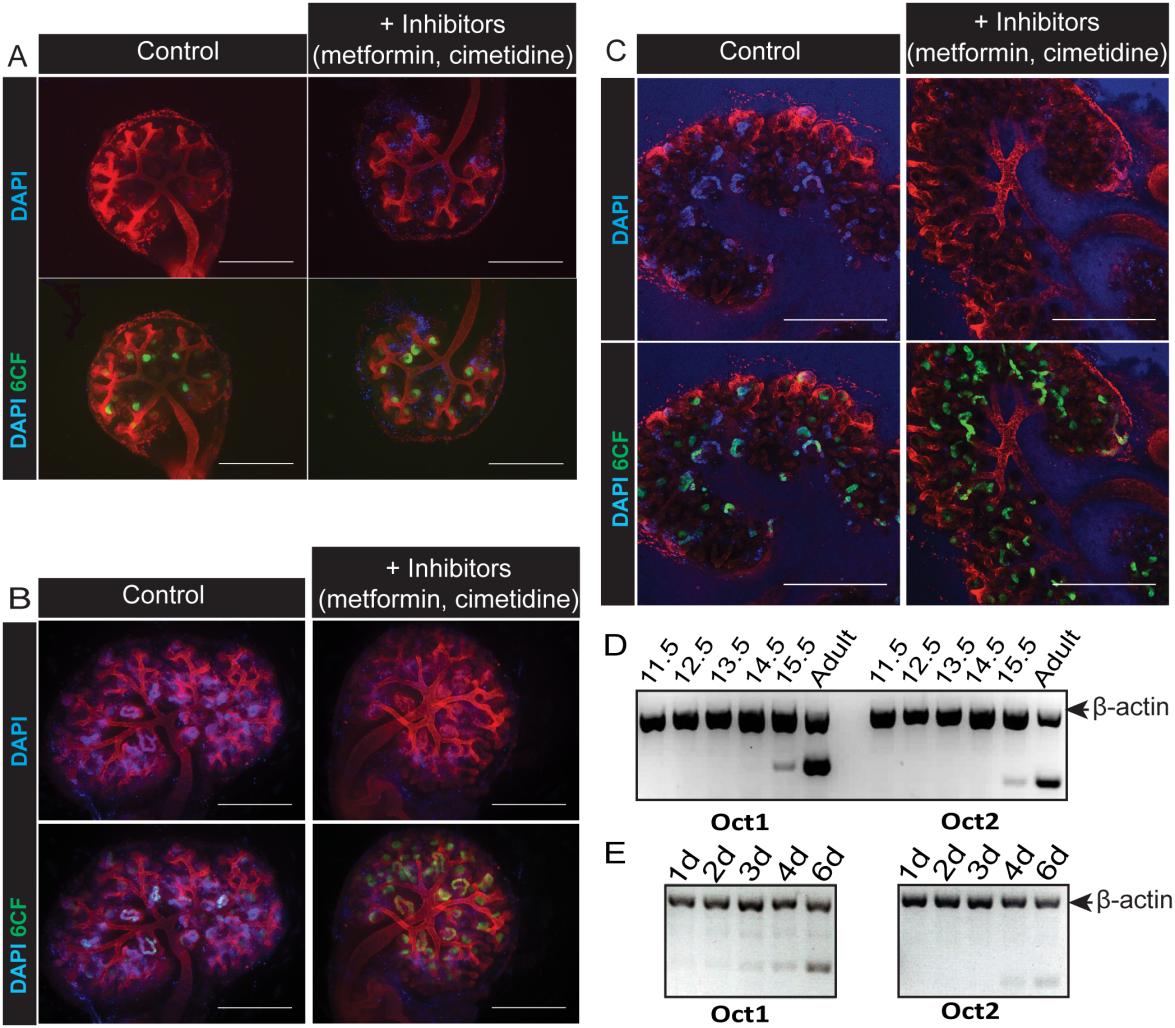
Onset of tubular organic cation uptake in developing kidneys in culture and in vivo. Specific uptake of the fluorescent cation DAPI with or without the Oct inhibitors metformin and cimetidine was investigated in E11.5 mouse kidneys grown in Sebinger culture after 3 days (A), 4 days (B) or 6 days (C) in culture. Tubules were also labelled by uptake of 6-CF (A–C, bottom panels). Cultures in (A) and (B) were imaged using an epifluorescence microscope. Cultures in (C) were imaged using a confocal microscope and images shown are of a single confocal plane. Scale bars represent 500 μm. D) Total mRNA was extracted from kidneys at various embryonic stages and from adult murine kidney. Detection of mRNA transcripts from two renal murine organic cation transporters was evaluated by multiplex RT-PCR (gene of interest and β-actin). (E) Total mRNA was extracted from E11.5 embryonic kidneys cultured for 1, 2, 3, 4, or 6 days. Detection of mRNA transcripts from two murine renal organic cation transporters was evaluated by multiplex RT-PCR (gene of interest and β-actin).

We assessed qualitatively the ability of embryonic kidneys, also cultured from E11.5, to specifically take up the fluorescent cation DAPI in the presence or absence of metformin and cimetidine, inhibitors of the organic cation family of transporters (Octs). DAPI does not normally penetrate intact membranes of live cells, but can be transported into cells expressing Oct1^31^. We found that uptake of DAPI was observed in the tubules of cultured embryonic kidneys after 4 days in culture (Figure 4B) and this functional characteristic of the tubules was maintained after 6 days in culture (Figure 4C) and beyond. In contrast, no specific tubular uptake of DAPI was observed up to 3 days in culture (Figure 4A). To demonstrate the presence of (functional) tubules in this assay, we used 6-CF, which is taken up through the anion channels and therefore unaffected by cation channel inhibitors (Figure 4A–C bottom panels).

The principal renal organic cation channels found in mice are Oct1 and Oct2^37^. Transcript analysis of Oct1 and Oct2 by semi-quantitative RT-PCR in embryonic kidneys allowed to develop *in vivo* and dissected at different embryonic time-points indicated that the transcripts for Oct1 and Oct2 are detectable at E15.5 (Figure 4D). In cultured kidneys, dissected at E11.5 and cultured for 1, 2, 3, 4 or 6 days, Oct1 transcripts were weakly detected from 3 days in culture and increased after 4 days in culture (Figure 4E). Oct2 transcript was weakly detected after 4 days in culture.

**Figure 5:**
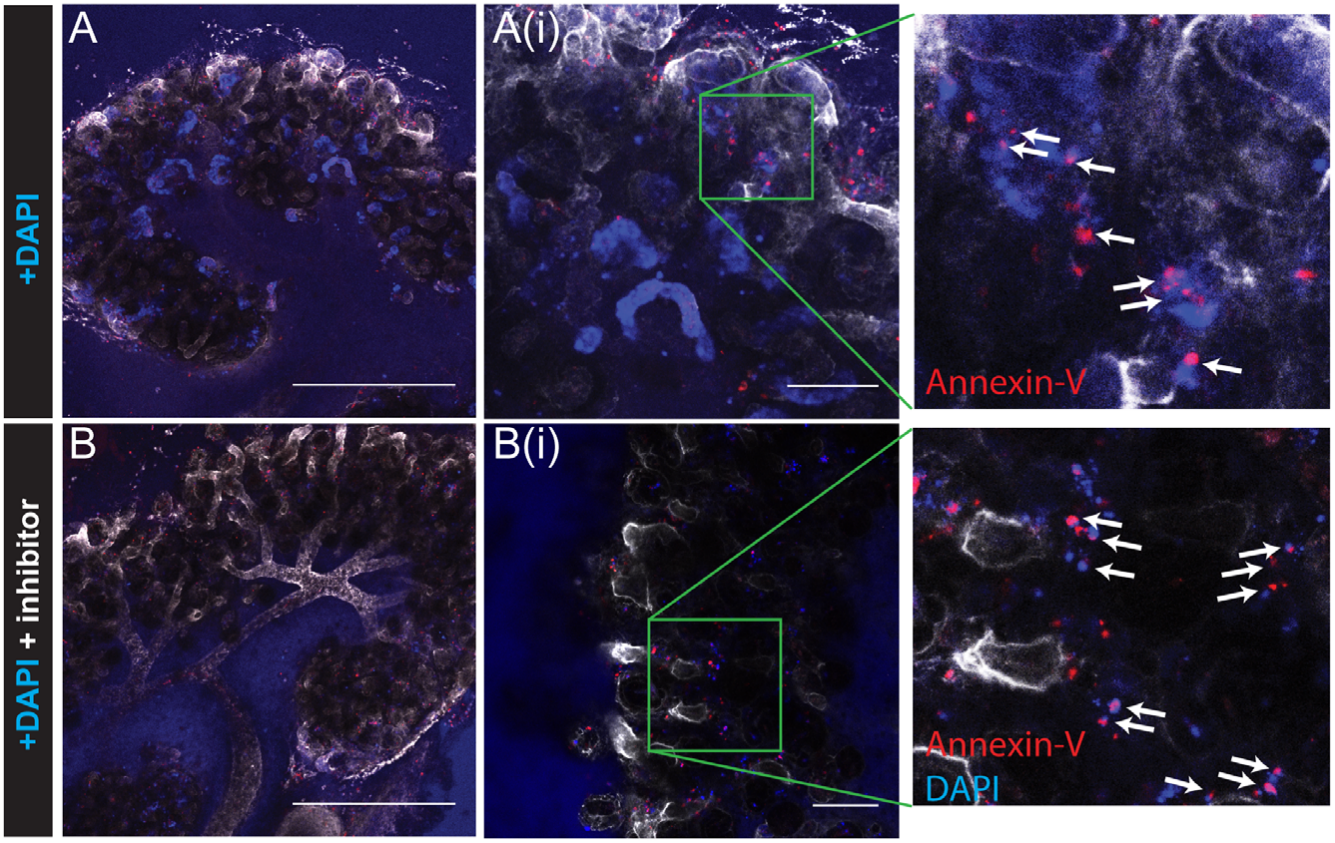
DAPI staining outside of tubules shows necrotic or apoptotic nuclei. Uptake of DAPI by Oct family cation transporters after 6 days in Sebinger culture either without (A) or with (B) Oct inhibitors metformin and cimetidine followed by staining with Cy5-conjugated Annexin-V. Annexin-V staining is associated with DAPI-positive apoptotic nuclei outside the tubules (A(i)) and B(i), and boxed enlargements white arrows), but not with DAPI-positive (non-apoptotic) tubular nuclei (Ai). Images were captured by confocal microscopy. Scale bars represent 500 μm (A, B), or 100 μm (A(i), B(i)).

Whilst there was clear tubular accumulation of DAPI in cultured kidneys by 4 days in culture, we observed a small amount of nuclear DAPI staining outside the tubules in all the kidneys, whether treated with inhibitor or not, and at all stages (Supplementary Figure S1(A)). We surmised that these might be apoptotic or dead cells with cell membranes no longer intact, allowing DAPI to penetrate and bind to the DNA. To test this, we used the phospholipid-binding protein Annexin-V, which has high affinity for phosphatidylserine (PS). Early in apoptosis, PS is translocated from the inner cell membrane to the outer. Necrotic cells also lose their membrane integrity and thus PS is also accessible to Annexin in these cells. Fluorescently labelled Annexin-V can therefore be used to identify apoptotic and necrotic cells^38^, so we used Annexin-Cy5 to identify cells whose membrane integrity had been lost (Figure 5). Almost all the cells that were DAPI-positive but not within tubules were also labelled for Annexin-V (shown in red), whether kidneys had been treated with uptake inhibitor (Figure 5B) or not (Figure 5A). In contrast, tubular cells that were positive for DAPI were not labelled with Annexin-V.

### Serially-reaggregated engineered kidneys exhibit some functional characteristics of intact cultured kidneys

This laboratory has previously shown that early murine kidney rudiments comprising only two tissue types, ureteric bud and metanephric mesenchyme, are capable of self-assembling into renal structures and progressing developmentally after dissociation into single-cell suspension^1^. Refinement of this technique by serial reaggregation and in Sebinger culture allows the engineering of a self-assembled kidney organised around a single collecting duct and with distinct structures such as loops of Henle, making them morphologically very similar to intact kidneys grown in culture^2^,^4^.

**Figure 6:**
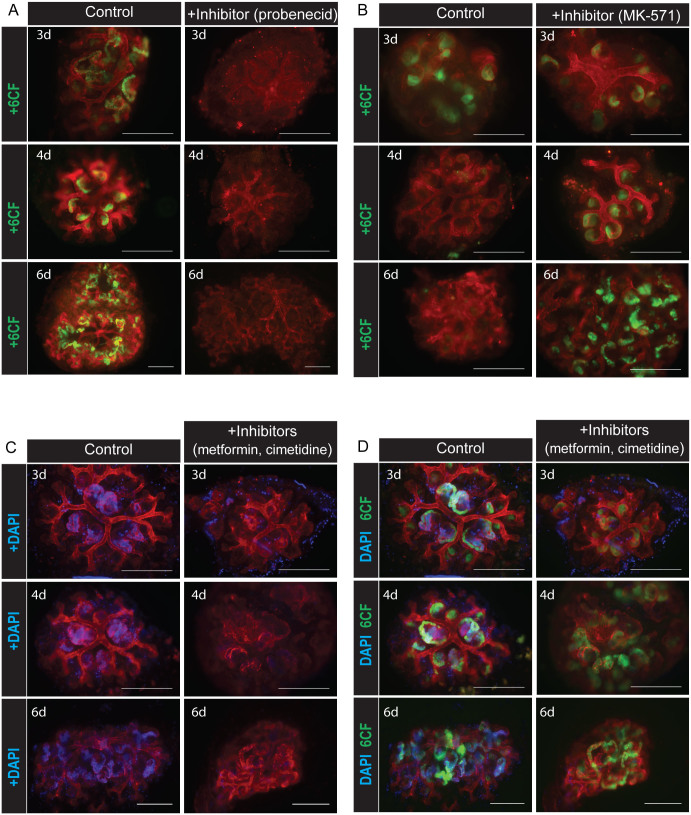
Tubules in serially-reaggregated engineered kidneys transport organic anions and cations. (A) Specific tubular uptake by organic anion transporters of fluorescent anion 6-CF in serially-reaggregated engineered kidneys cultured for 3, 4 or 6 days after addition of mesenchyme. (B) Specific tubular efflux by the Mrp family of efflux proteins of the fluorescent anion 6-CF in serially-reaggregated engineered kidneys cultured for 3, 4 or 6 days after addition of mesenchyme. (C) Specific tubular uptake by organic cation transporters of fluorescent cation DAPI in serially-reaggregated engineered kidneys cultured for 3, 4 or 6 days after addition of mesenchyme. (D) Tubular uptake of 6-CF by organic anion transporters in the serially-reaggregated engineered kidneys shown in (C), demonstrating presence and functional ability of tubules in these serial reaggregates both with and without the organic cation transporter inhibitors. Scale bars represent 300 μm.

We used the transport assays described above to investigate whether the tubular epithelia in serially-reaggregated engineered kidneys are functional. Number of days in culture was counted beginning from addition of fresh, dissociated mesenchyme to isolated, previously dissociated and now reaggregated ureteric bud cysts (see Materials and Methods). Specific uptake of the fluorescent anion 6-CF by organic anion transporters was observed from 3 days in culture, with no detectable uptake in serially reaggregated, engineered kidneys treated with the Oat inhibitor probenecid (Figure 6A). Specific efflux of 6-CF through the Mrp-family of efflux pumps was also observed from 4 days in culture (Figure 6B), with no efflux seen after only 3 days in culture, or in cultures treated with the Mrp-specific inhibitor MK-571. Finally, we investigated whether the tubular epithelia of serially-reaggregated, engineered kidneys were capable of cation transport. Cultures were treated with the fluorescent cation DAPI with or without the presence of the cation inhibitors metformin and cimetidine. We observed uptake of the fluorescent cation DAPI into the tubules from 3 days in culture, but no uptake was observed when the serially-reaggregated engineered kidneys were treated with the Oct inhibitors metformin and cimetidine (Figure 6C). To demonstrate the presence of (functional) tubules in the serially-reaggregated kidneys assayed for cation transport function, tubules were also labelled with 6-CF (Figure 6D).

## Discussion

Our results demonstrate that tubular transport function begins early in kidney development in agreement with previous microarray analyses^13^, and that cultured embryonic kidneys behave in a similar way to kidneys allowed to develop *in vivo* with respect to tubular function. In addition, we have shown that serially-reaggregated engineered kidneys also develop this transport function. Whilst basolateral to luminal transport is an important component of tubular function, tubules also transport molecules from the tubule lumen that have passed through the glomerular filtration barrier, and we have not yet determined if this process is active in cultured or serially-reaggregated engineered kidneys.

We have shown that transport of organic anions and cations is not dependent on development of vasculature and glomerular filtration. There are other examples of the uncoupling of tubular transport and blood filtration in development, for example in insects, where the functions take place in different locations in the body^39^,^40^. The purpose of basolateral to luminal transport early in the development of the embryo is unknown: it may provide an early excretory route for endogenous compounds and xenobiotics.

Previous work has suggested that anatomically-correct, reaggregated kidneys are able to develop vasculature and to develop some degree of glomerular filtration^41^. This study provides evidence that serially-reaggregated engineered kidneys can also develop the critically-important function of active transport, making the prospect of developing engineered kidneys for clinical use or drug screening more relevant.

## Methods

### Embryonic Organ Culture

Embryonic kidneys were dissected from E11.5 mouse embryos and placed in Sebinger culture^3^, in which they were cultured for up to 10 days. Kidney cultures were incubated in 82–85 μl kidney culture medium (KCM) (KCM consists of Eagle's MEM with Earle's salts (Sigma, M5650) supplemented with 10% fetal calf serum (Invitrogen, 10108165) and 1% Penicillin/Streptomycin (Sigma, P4333)). Medium was changed daily.

### Formation of Serial Reaggregates (Engineered kidneys)

Serial reaggregates were engineered according to the method described in refs. 1, 2. Briefly, 8–10 kidneys were dissected from E11.5 mouse embryos and pooled. These were trypsinised for 1 min at 37°C before manually dissociating and passing twice through a cell strainer (40 μm pore size). The dissociated cells were pelleted by centrifuging at 9000 × g and then cultured in conventional organ culture system (Isopore filters supported by metal grids) in KCM. For the first 24 hours media was supplemented with the ROCK inhibitor glycyl-H1152-dihydrochloride (1.25 μM). After 3–4 days in culture, single ureteric bud cysts were isolated by dissection and either placed in conventional organ culture, or placed in Transwell dishes (using Greiner CELLSTAR® 6 well plates), and surrounded by dissected, dissociated and pelleted mesenchyme from 8–10 new E11.5 kidneys. After 24 hours, the aggregated tissue was placed into Sebinger culture and grown for a further 3, 4 or 6 days.

### Chemicals and Reagents

The following reagents were purchased from Sigma Aldrich; Metformin hydrochloride (PHR1084), Cimetidine (C4522), Probenecid (P8761), MK-571 (M7571), Digoxin (D6003). 6-carboxyfluorescein was purchased from Invitrogen (C1360). Glycyl-H 1152 dihydrochloride was purchased from Tocris (2485). Metformin, cimetidine, and 6-carboxyfluorescein were solubilised in water to make stock concentrations as follows: metformin 100 mM, cimetidine 25 mM, 6-carboxyfluorescein 1 mM. Probenecid was solubilised in 500 mM NaOH, to make a stock concentration of 250 mM. MK-571 and digoxin were solubilised in DMSO to make stock concentrations as follows: MK-571 10 mM, digoxin 93.5 mM. Reagents for live assays were diluted in PBS.

### Transporter Assays

All assays were carried out on live cultures grown in the Sebinger culture system. Only cultures that were healthy at the time of assay were used; for intact, cultured kidneys this was approximately 8 in 10. For serially reaggregated engineered kidneys this number dropped to approximately 6 in 10. Live staining to outline morphological structure was done in all cases by incubating cultures, at same time as the transporter assay, with rhodamine-conjugated peanut aggulutinin (20 μg/ml Vector Laboratories, RL-1072). Prior to assay all cultures were washed once in PBS. Specific uptake of anions in the tubules of cultured kidneys was assayed by incubating live cultures with the fluorescent anion 6-carboxyfluorecein (6-CF) (1 μM) either with probenecid (2.5 mM), or with vehicle only (final concentration of NaOH for both conditions, 5 mM) for one hour at 37°C. Cultures were washed once in PBS and then incubated with probenecid (10 mM) for 15 min at 37°C to trap intracellular fluorophore before imaging. Anion uptake assays in cultured intact kidneys were repeated 3 times (1 d), twice (2 d) or 7 times (6 d). Uptake in untreated kidneys and inhibition of uptake in inhibitor-treated kidneys was evident in every case. Specific efflux of anions by the Mrp family of efflux pumps was assayed by pre-loading live cultures with 6-CF for one hour at 37°C (1 μM), washing once in PBS and imaging briefly to confirm anion uptake, and then subsequently incubating with either digoxin (500 uM) and vehicle (0.33% DMSO), or digoxin (500 μM) and MK-571 (30 uM) (final DMSO concentration for both conditions 0.83%), for 30–60 min at 37°C. Anion efflux assays in cultured intact kidneys were repeated twice (2 d, 3 d) or 4 times (4 d, 6 d) with efflux in kidneys untreated with MK571, and inhibition of efflux in MK571-treated kidneys evident in every case. Specific uptake of cations in the tubules of cultured kidneys was assayed by incubating live cultures with the fluorescent cation DAPI (1 μM) alone or in the presence of metformin (5 mM) and cimetidine (200 μM) for 30 minutes at 37°C. Cultures were washed 3x in PBS before imaging. Because DAPI is bound stably to DNA once inside a cell, the cultured kidneys could be imaged directly without needing to trap the fluorophore intracellularly. Cation uptake assays in cultured intact kidneys were repeated twice (3 d, 4 d) or 10 times (6 d). Uptake in untreated kidneys and inhibition of uptake in inhibitor-treated kidneys was evident in every case. All assays (uptake and efflux) in serially-reaggregated kidneys were performed once (3 d, 4 d) or twice (6 d). Inhibition of uptake or efflux in inhibitor-treated cultures, and no inhibition of uptake or efflux in untreated cultures, was evident in every case.

### Annexin-V staining of live organ cultures

An AnnexinV assay kit was purchased from Biovision (K103-25). After carrying out the cation transporter assay, live cultures were washed 3x in PBS and then incubated with AnnexinV binding buffer for 5 min at room temperature. Cultures were then incubated with AnnexinV solution diluted 1:100 in binding buffer at 37°C. Cultures were imaged immediately by confocal microscopy.

### Imaging

Live cultures were imaged using a Zeiss Axiovert epifluorescence microscope with Axiovision software, or using a Nikon A1R confocal microscope with NIS elements software. When applicable all images were taken with identical exposures for comparison. All images were analysed using ImageJ.

### Isolation of mRNA and RT-PCR

Total mRNA was isolated from 25–30 pooled embryonic kidneys, pooled cultured kidneys, or adult kidney using the RNeasy kit from Qiagen and according to kit instructions. After isolation, purity of RNA and absence of genomic DNA was confirmed by PCR for β-actin using primers that span an intron and using RNA preparations made with and without addition of reverse transcriptase. PCRs for genes of interest were carried out in multiplex reactions with primers for β-actin as loading controls. A table of primers used can be found in the supplementary information, Table 1.

### Animals

This project involved no experiments conducted in living animals. Normal embryonic tissues used for post-mortem staining and culture were obtained from healthy CD1 mice killed by trained staff of the UK Home Office-licenced animal house under Schedule 1 of the UK Animals (Scientific Procedures) Act 1986.

## Author Contributions

M.L. performed all experiments, drafted the manuscript and prepared the figures. C.C. prepared RNA from cultured kidneys and helped to make the serial reaggregates. J.D. contributed to experimental design and data analysis, and edited the manuscript. All authors reviewed the manuscript.

## Supplementary Material

Supplementary Information Supplementary info
