# Supplementary material for: Transport of organic anions and cations in murine embryonic kidney development and in serially-reaggregated engineered kidneys
Source: Sci Rep. 2015 Mar 13;5:9092. doi: 10.1038/srep09092 (PMC4357899; doi:10.1038/srep09092)
Supplement: Supplementary Information — Supplementary info [file srep09092-s1.pdf]

Transport of organic anions and cations in murine embryonic kidney development and in serially-reaggregated engineered kidneys

Melanie L Lawrence, C-Hong Chang, Jamie A Davies*
Centre for Integrative Physiology, The University of Edinburgh

Supplementary Figure S1

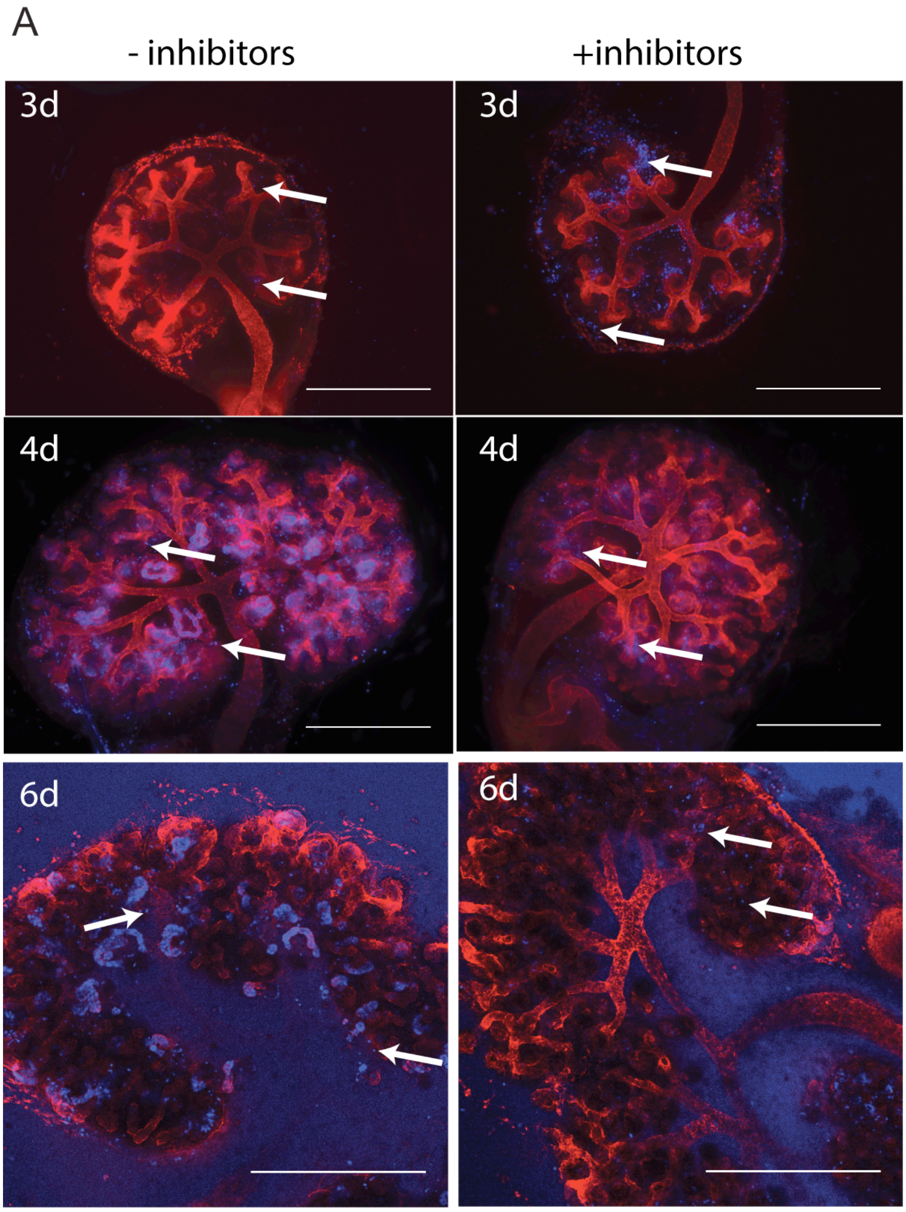

Figure S1. *DAPI-positive nuclei outside tubules in cultured embryonic kidneys*

(A) White arrows point to examples of DAPI-positive cells (blue) that are not inside tubules in live cultured kidneys, at 3 days, 4 days and 6 days in culture and both with and without inhibitor treatment. Scale bars represent 300um.

Supplementary Table S1

Primers for RT-PCR

**Oat1**
Forward primer: ATGGTGGGAGTGTTACTGGG
Reverse primer: GGAGCCGGAAAATGCAGTAG
**Oat3**
Forward primer: TACAGTTGTCCGTGTCTGC
Reverse primer: TTCAGCTCCTCCACAGTGAG
**Oatp4c1**
Forward primer: TTATGATAGGCCTGGGAGCG
Reverse primer: CCAGCAACAATTGTCCCAGG
**Oct1**
Forward primer: GTCCTTCGTTTGCAGACCTG
Reverse primer: TATTGGGTAGATGCGGCCA
**Oct2**
Forward primer: TGGGCATTGGTTACCTAGCA
Reverse primer: TTGCTGACCAGTCCCTGTAG
**Mrp2**
Forward primer: ACACCAACCAGAAATGCGTC
Reverse primer: GGACAGAACAAAGCCCACAG
**β -actin**
Forward primer: CTGGGACGACATGGAGGARA*
Reverser primer: AAGGAAGGCTGGAARAGWGC*

- R=50:50 A+G, W=50:50 A+T
